# Data-Driven Fault Detection and Diagnosis in Cooling Units Using Sensor-Based Machine Learning Classification

**DOI:** 10.3390/s25123647

**Published:** 2025-06-11

**Authors:** Amilcar Quispe-Astorga, Roger Jesus Coaquira-Castillo, L. Walter Utrilla Mego, Julio Cesar Herrera-Levano, Yesenia Concha-Ramos, Erwin J. Sacoto-Cabrera, Edison Moreno-Cardenas

**Affiliations:** 1 LIECAR Laboratory, Universidad Nacional de San Antonio Abad del Cusco (UNSAAC), Cusco 08003, Peru; 134552@unsaac.edu.pe (A.Q.-A.); roger.coaquira@unsaac.edu.pe (R.J.C.-C.); 2 TESLA Laboratory, Universidad Nacional de San Antonio Abad del Cusco (UNSAAC), Cusco 08003, Peru; walter.mego@unsaac.edu.pe (L.W.U.M.); julio.herrera@unsaac.edu.pe (J.C.H.-L.); 3 Professional Academic School of Systems and Computer Engineering, Universidad Continental, Cusco 08000, Peru; yconcha@continental.edu.pe; 4 GIHP4C, Universidad Politécnica Salesiana, Cuenca 010102, Ecuador; esacoto@ups.edu.ec; 5 Communications Department, Universitat Politècnica de València, 46022 Valencia, Spain

**Keywords:** system cooling unit, fault detection and diagnosis, machine learning, sensors, data-driven

## Abstract

Precision air conditioning (PAC) systems are prone to various types of failures, leading to inefficiencies, increased energy consumption, and possible reductions in equipment performance. This study proposes an automatic real-time fault detection and diagnosis system. It classifies events as either faulty or normal by analyzing key status signals such as pressure, temperature, current, and voltage. This research is based on data-driven models and machine learning, where a specific strategy is proposed for five types of system failures. The work was carried out on a Rittal PAC, model SK3328.500 (cooling unit), installing capacitive pressure sensors, Hall effect current sensors, electromagnetic induction voltage sensors, infrared temperature sensors, and thermocouple-type sensors. For the implementation of the system, a dataset of PAC status signals was obtained, initially consisting of 31,057 samples after a preprocessing step using the Random Under-Sampler (RUS) module. A database with 20,000 samples was obtained, which includes normal and failed operating events generated in the PAC. The selection of the models is based on accuracy criteria, evaluated by testing in both offline (database) and real-time conditions. The Support Vector Machine (SVM) model achieved 93%, Decision Tree (DT) 93%, Gradient Boosting (GB) 91%, K-Nearest Neighbors (KNN) 83%, and Naive Bayes (NB) 77%, while the Random Forest (RF) model stood out, having an accuracy of 96% in deferred tests and 95.28% in real-time. Finally, a validation test was performed with the best-selected model in real time, simulating a real environment for the PAC system, achieving an accuracy rate of 93.49%.

## 1. Introduction

Precision air conditioning (PAC) systems are critical infrastructure for environmental control in environments where thermal stability and air quality are essential, such as data centers, biomedical laboratories, hospital facilities, and the telecommunications and electronics manufacturing industries [1,2]. Unlike conventional HVAC systems (heating, ventilation, and air conditioning), PACs are designed to operate continuously under demanding conditions, managing minimal thermal variations with high efficiency and reliability. This need for sustained and controlled operation introduces greater complexity in their structure, as they are composed of interdependent components such as scroll compressors, thermostatic expansion valves, condensers, and evaporators that are exposed to mechanical degradation processes, dirt accumulation, corrosion, and failures induced by refrigerant overcharge or undercharge [3].

In this sense, the timely detection of faults in PAC systems is essential not only to ensure their correct operation, but also to avoid critical outages, increase their lifetime, and reduce energy consumption. However, traditional approaches to Fault Detection and Diagnosis (FDD)—based on manual inspections, corrective maintenance, or expert systems with fixed thresholds—have serious limitations with respect to efficiency, scalability, and adaptability, as described in [4]. In addition, these methods are often dependent on the experience of the operator and cannot be learned or adapt to changing conditions. These limitations are exacerbated in industrial environments where systems must operate without interruption and under remote supervision.

In this context, data-driven FDD approaches have emerged as a promising alternative that allows the automation of the diagnostic process from sensor signals such as pressure, temperature, current, and voltage using Machine Learning (ML) techniques to identify anomalous patterns in real time [5,6]. ML-based methods have proven to be effective in HVAC systems, far outperforming physical-model-based approaches, which require detailed characterization of each system and are often inflexible in the face of structural or dynamic variability, as described in [7,8].

In the same sense, the authors in [9] have shown that supervised models such as SVM, Random Forest (RF), Gradient Boosting (GB), and K-Nearest Neighbors (KNN) can achieve accuracies above 90% in the detection of faults in rooftop air conditioning units. However, the different studies have focused on specific or simulated configurations, leaving a gap in the practical implementation of embedded diagnostic systems with real-time inference and validation capabilities in real PAC units.

This work addresses the limitations described above by developing an automatic and embedded FDD system for PAC, leveraging a distributed acquisition and processing architecture using an Arduino and Raspberry Pi fed by real-time data from physical sensors and validated under real operating conditions. A classification methodology is proposed for five common fault types—Refrigerant Undercharge (RU), Refrigerant Overcharge (RO), Line Restriction (RL), Condenser Airflow Reduction (CA), and Evaporator Airflow Reduction (EA)—combining a robust preprocessing, including the use of RandomUnderSampler (RUS) with a comparative analysis of six classification algorithms: SVM, NB, DT, KNN, GB, and RF.

The central objective of this work is to identify the most effective ML model for accurate and efficient fault diagnosis in PAC systems, both in offline testing and online implementation, maximizing accuracy, sensitivity, and specificity while ensuring feasibility in embedded environments with limited computational resources.

This article presents the following contributions.

- Develop a comprehensive database for training FDD models by capturing the behavioral patterns of five types of faults, RU, RO, RL, CA, and EA, based on critical state signals such as pressure, temperature, current, and voltage.
- Evaluate and compare the performance of multiple ML classification models, including SVM, KNN, DT, GB, NB and RF, to determine the most effective algorithm for fault detection in PAC units.
- Implement and validate the FDD system in real-time conditions, optimizing hyperparameters through GridSearchCV, identifying the most influential predictor variables (Tcomp, Icomp, Vcomp, Wcomp), and evaluating the viability of the system for the predictive maintenance of HVAC systems in real environments, focusing on operational efficiency, downtime reduction, and maintenance cost savings.

Compared to previous studies, this work presents three novel elements: (i) the implementation and validation of an FDD system under real-world conditions using an industrial PAC system (model SK3328.500), (ii) the use of a low-cost embedded architecture based on Arduino Nano and Raspberry Pi for real-time processing, and (iii) a comprehensive comparative approach using six ML classification models with the analysis of specific metrics such as accuracy, sensitivity, specificity, and false positive rate. This practical integration of sensing, local processing, and automated diagnosis, with validated results in real-world environments, represents a concrete contribution to the effective implementation of intelligent predictive maintenance systems in industrial HVAC systems.

Finally, the proposed system adopts a methodology structured in five stages: (1) data acquisition using sensors installed in a real PAC unit; (2) preprocessing with cleaning and balancing techniques such as RandomUnderSampler; (3) feature extraction from pressure, temperature, current, and voltage signals; (4) the training of supervised models with libraries such as Scikit-learn; and (5) real-time validation on an embedded architecture composed of Arduino Nano and Raspberry Pi 4. The integration of specific sensors (ACS712, ZMPT101B, SPKT0043P, DS18B20, and MLX90614), together with a MySQL database and local processing, enables a robust, scalable, and reproducible solution for automated FDD in industrial HVAC systems.

### Related Work

The current literature on FDD in HVAC systems revolves around four main approaches: (i) data-driven methods, (ii) classification algorithms using ML, (iii) data preprocessing and data balancing techniques, and (iv) hybrid strategies that integrate physical rules with ML in embedded implementations. In this section, we review these approaches, identifying the strengths and limitations of previous work and highlighting the gaps that the present study aims to address. In this sense, data-driven methods have proven to be an effective alternative to physical models, as they allow the analysis of large volumes of sensor data without the need to model the system’s behavior mathematically. For example, the authors in [1] present a systematic review on the use of computational intelligence for FDD, highlighting how statistical approaches have given way to the use of big data and ML techniques. Furthermore, regarding practical applications, in [4] the authors propose an FDD system for chillers using supervised learning, obtaining accuracies of over 95%. However, in [4], the authors developed their method under laboratory conditions and do not consider aspects such as the unbalanced distribution of faults in real scenarios.

In the framework of the use of classification algorithms, different models, such as SVM, RF, and GB, have been compared; it has been concluded that RF achieves the best results in terms of accuracy and robustness. However, these studies usually omit alternative classifiers such as KNN or NB, as well as not including the importance analysis of predictor variables. Furthermore, the treatment of input data has been identified as a critical factor for the success of FDD. For example, the authors of [6] employ virtual sensors to improve signal fidelity, although without comprehensively addressing the problem of class imbalance. In addition, some studies have implemented basic oversampling techniques to mitigate this effect, but without robust validation under real operating conditions [10]. In contrast, the present study uses a controlled undersampling approach (RUS) to obtain a balanced dataset, thus ensuring a representative distribution of all types of failures.

Finally, in relation to hybrid systems and real-time applications, solutions combining physical rules with ML algorithms have been proposed, as in [11], where a hybrid architecture based on RF and SVM is used. However, most of these works do not implement complete embedded solutions and do not offer real-time explainable diagnostics. On the other hand, ref. [5] demonstrates that electrical signals such as current and voltage can be successfully employed for basic FDD in rooftop units but cannot be extended to more structurally complex PAC systems.

Considering what has been described in the previous paragraphs, the present work differentiates itself by integrating multiple classifiers, applying a detailed analysis of critical features, and developing a real-time embedded system that allows both the detection and automated diagnosis of faults in industrial HVAC systems to seeking to close some of the technical and methodological gaps present in the described state of the art.

The remainder of this article is structured as follows. Section 2 presents and describes the system model and its structure, divided into two parts: an experimental system and an acquisition and processing system. Section 3 describes the research methodology, which addresses data preparation and processing, supervised models, and proposed rules-based diagnosis. Section 4 summarizes the main research results, comparing the performance of six ML models. Section 5 presents a discussion of our results in comparison with the results of previous studies. Section 6 describes the overall conclusions of the study. Finally, Section 7 suggests future research avenues that are opened up by this study.

## 2. System Model

**Figure 1:**
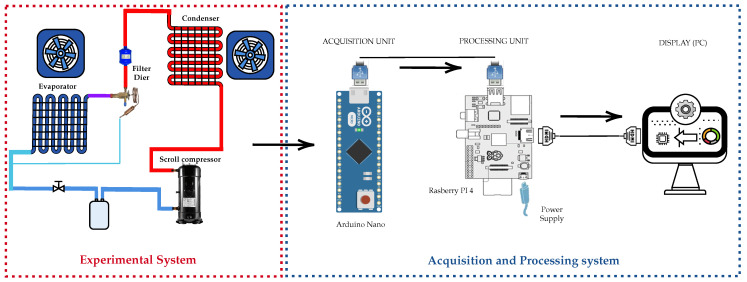
System model.

The overall system model is composed of two systems, as shown in Figure 1. First, an experimental system comprises a PAC, a refrigeration unit, and sensors, and second, a data acquisition and processing system consisting of an acquisition unit, a processing unit, and a graphical display interface. The proposed structure implements a three-component architecture working in synchrony. First, the microcontroller acquisition unit captures the critical variables of the PAC system in real time. Then, the captured signals are transmitted to a central processing unit where ML algorithms analyze the patterns to detect and classify different types of characteristic faults. Finally, the diagnostic results are displayed on a graphical interface, completing the workflow from data acquisition to automated diagnostic visualization. These parts are described in Section 2.1, Section 2.2 and Section 2.3.

### 2.1. Experimental System

**Figure 2:**
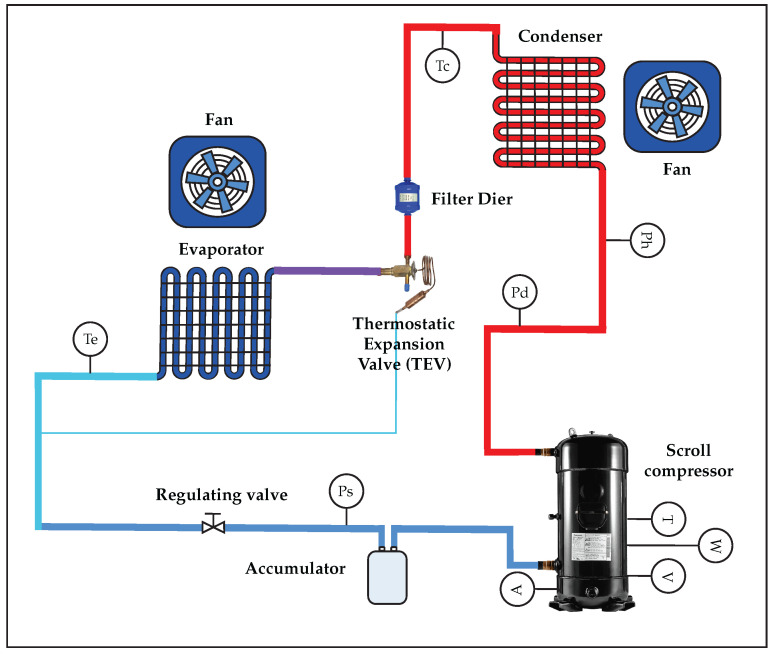
Experimental system with the measurement sensors: Compressor Current (A), Compressor Voltage (V), High-Pressure Line (Ph), Discharge Pressure (Pd), Evaporator Temperature (Te), Condenser Temperature (Tc), Compressor Temperature (T), Compressor Power (W), and Suction Pressure (Ps).

The experimental system consists of a PAC system, a refrigeration unit, and the main sensors, as shown in Figure 2. The PAC system uses a hydrofluorocarbon refrigerant gas (R134A) as a refrigerant and a nominal charge of 9.9 kg with a nominal capacity of 2 kW, respectively, whose values are described in Table 1. The flow status in the refrigeration cycle is represented by the red lines representing the high-pressure pipes, while the blue lines represent the opposite.

The fault diagnosis and detection evaluates five types of faults. These faults are shown in Table 2 and described below.

- **Refrigerant overcharge (RO):** The operation of this equipment is designed for low pressure, so an excess of refrigerant increases the pressure and temperature, reducing its cooling capacity [10,12].
- **Refrigerant undercharge (RU):** This occurs due to leaks or poor maintenance. Thus, a certain pressure must be maintained on the low-pressure side for optimum performance. However, insufficient refrigerant reduces the system’s operating pressure and temperature, decreasing its cooling capacity [10,13].
- **Restriction in the liquid line (RL):** Caused by kinks or clogged filters in the liquid line [5]. This restriction leads to the accumulation of corrosive deposits and microorganisms that clog the pipes and cause damage to the equipment.
- **Condenser airflow reduction (CA):** Caused due to dirt on the heat exchanger (heatsink), insufficient airflow from the condenser fan, or design problems in the distribution system [10].
- **Evaporator airflow reduction (EA):** Caused due to factors similar to AC.

The selection of the five failure types (RU, RO, RL, CA, EA) was based on the frequency criticality observed in PAC systems installed in mission-critical environments such as data centers and laboratories; these failures are the most common and directly affect the operational efficiency and thermal performance of the system. Other types of potential failures, e.g., internal valve leaks or liquid backflow, were not included due to instrumental constraints, the limited availability of the system to induce failures in a controlled manner, and because they are beyond the scope of this first experimental phase.

Table 3 shows some statistical descriptors of the input variables of the total dataset, which are the main signals of the PAC system. Based on [3,14], the following measurements were selected. (1) Pressure (Ph) in the high-pressure line, (2) suction pressure (Ps), (3) discharge pressure (Pd). With these pressure measurements, the behavior in the main stages of the system is evaluated, where low- and high-pressure values are recorded within the same system for failure events and those of the compressor. (4) Temperature (T°) at the evaporator outlet, (5) temperature (T°) at the compressor outlet, (6) contact temperature (T°) of the compressor casing. These measurement evaluates the behavior in cases of failure events under the principle that heat generation, which is an unavoidable by-product of the operation of most systems, is generated by several factors. This phenomenon is generated by factors such as suboptimal operating conditions, which produce an excessive temperature rise. (7) Compressor working current (I), (8) compressor voltage (V), and, finally, (9) compressor power (W). With these last measurements, the variation in power consumption in situations of system failure events is evaluated.

### 2.2. Acquisition and Processing System

**Figure 3:**
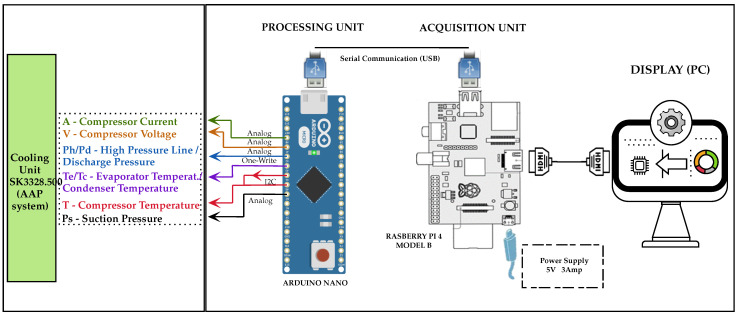
Acquisition and processing system.

**Figure 4:**
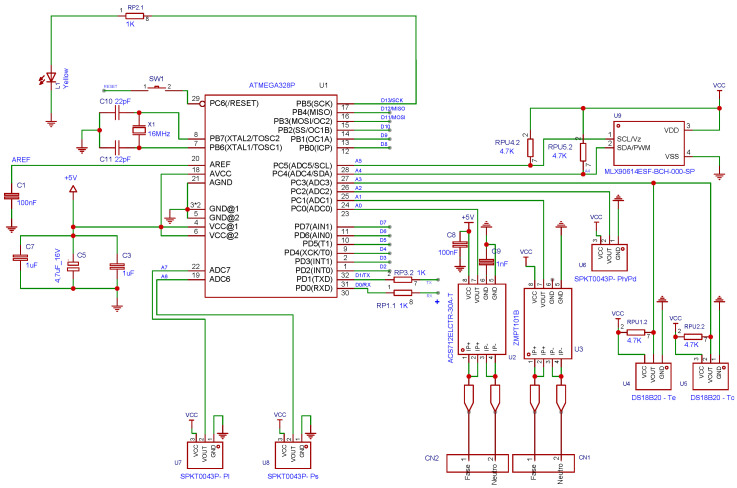
Instrumentation circuit schematic diagram (acquisition system).

The acquisition and processing system is designed to ensure accurate and real-time detection in the refrigeration unit (sk3328.500), as shown in Figure 3. The design of this system [15] has a two-stage architecture: an Arduino for signal acquisition and a Raspberry Pi as the main processing unit. Arduino was selected for its low power consumption, compatibility with sensors, and support for different protocols, such as I2C, which captures temperature, pressure, current, and voltage data. This data is transmitted via USB to the Raspberry Pi, which stores it in a MySQL database for further analysis. Real-time processing uses ML algorithms, and the Raspberry Pi was chosen for its computing power, Linux environment, and support for Python libraries. The scope of real-time data processing encompasses feature extraction, anomaly detection, and fault classification. This modular architecture improves scalability, reliability, and integration with cloud monitoring systems and is adaptable to larger-scale HVAC applications. A detailed schematic of the instrumentation circuit and its connections is presented in Figure 4.

### 2.3. Overview of Data

**Figure 5:**
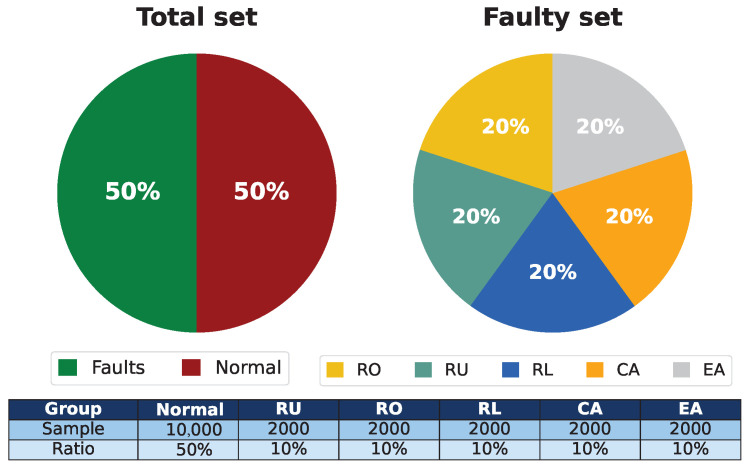
Representative diagram of each data set.

The acquisition system collects the data and transmits it to the microprocessor. Nine features are selected to build the total dataset, divided into a normal set and a failed set based on [3], labeling the latter as faults, while the failed set comprises five specific fault types. Figure 5 shows the distribution of the samples, where the normal samples represent 50% of the total set, while the RU, RO, RL, CA, and EA faults are equally distributed with 10% each in order to balance the data.

## 3. Methodology

**Figure 6:**
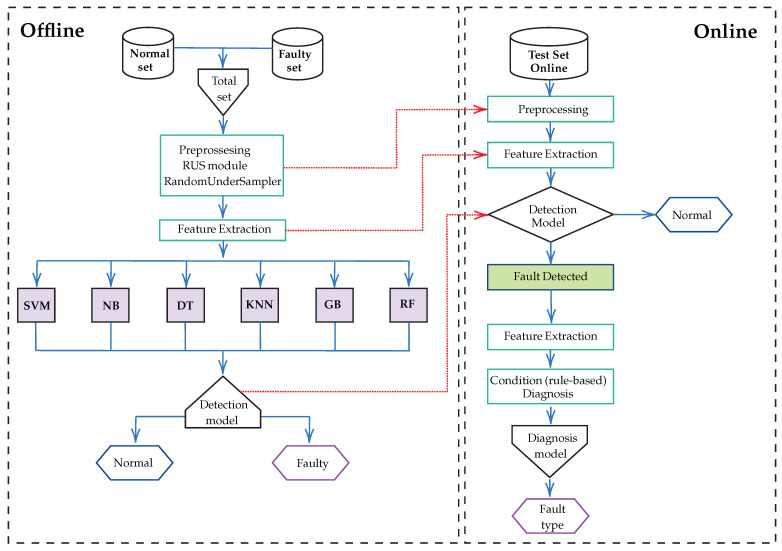
Framework of the proposed FDD strategy.

This study proposes a data-driven FDD strategy designed to address five specific types of failures [16]. Figure 6 shows the comprehensive strategic framework developed in this work, which guides the FDD methodology throughout this chapter. The framework begins with an offline phase to build a database of normal and faulty conditions. Then, the data is preprocessed in real-time in the online phase, e.g., with RUS, to balance the classes, and key features such as pressure, temperature, current, and voltage are extracted. In addition, six supervised learning models—SVM, NB, DT, KNN, GB, and RF [17]—are evaluated to select the optimal one. If the system detects a fault, a rule-based diagnostic module identifies the cause, for example, a refrigerant overcharge or undercharge, a line restriction, or reduced airflow in the condenser/evaporator [3,5]. This approach improves the accuracy and efficiency of fault detection, providing a robust system for monitoring and maintenance, as shown in Figure 6.

### 3.1. Data Preparation and Processing

Preprocessing and data preparation are performed to improve the reliability and accuracy of the proposed FDD framework, as the input dataset often contains incorrect, missing, inconsistent, or irrelevant samples, which affects the model’s ability to learn specific patterns. The initial dataset consists of more than 31,057 samples, so a thorough cleaning is performed to ensure that the model is trained with an unbiased and high-quality dataset. The unbalanced datasets are analyzed using a combination of sampling techniques to adjust the class distribution [10,11].

Oversampling methods are used to increase the representation of minority classes, and undersampling techniques are used to reduce the prevalence of majority-class samples. Therefore, in this study, preprocessing included a temporal analysis to remove observations unrelated to the cooling cycle, followed by the application of Scikit-learn’s RUS.fit_resample() method to balance unbalanced datasets by reducing the number of majority-class samples by randomly selecting them, which helps prevent ML models from biasing towards the most frequent classes [3].

### 3.2. Supervised Models

The supervised learning models used are based on the characteristics of their learning algorithms, as each of them is effective in FDD because of their ability to learn patterns from labeled data and generate accurate predictions [18]. Table 4 shows a confusion matrix structure to measure the performance of these models [19].

The effectiveness of the data-driven methods is evaluated using six supervised classification algorithms from the models, SVM, KNN, DT, GB, NB, and RF [20,21,22] on the generated and labeled databases containing normal operation and five defined failure modes. The main purpose is to address two fundamental questions. (1) Which classification method demonstrates the highest performance for this specific task? (2) What is the overall potential of data-driven approaches for real-time FDD in refrigeration systems? Each classifier is trained and validated using the same dataset under consistent conditions to ensure comparability. All statistical models are implemented using the Scikit-learn framework, a robust and widely adopted Python library for ML applications [7,18]. The specific functions and features of each model are summarized in Table 5 and described in the following sub-subsections. This systematic evaluation allows an objective comparison of classification accuracy and model behavior. This comparison provides a solid basis for selecting the most suitable algorithm for real-time implementation in HVAC systems [23].

#### 3.2.1. SVM Model

The SVM model searches for an optimal hyperplane that divides the data into two distinct categories internally, maximizing the margin between them [24,25]. The SVM model constructs an optimal hyperplane as follows:

(1) $$\begin{array}{c} W\cdot X+b=0 \end{array}$$

where *W* represents the vector of weights and defines the slope of the hyperplane, *X* is the vector of sample features, and *b* is the model bias and determines the hyperplane offset. On the other hand, the SVM model maximizes the margin between the classes.

(2) $$\begin{array}{c} M=\frac{2}{\left||W|\right|} \end{array}$$

where *M* is the separation between the hyperplane and the nearest points of each class, called support vectors. To handle cases where the data is not linearly separable, a regularization term is introduced in the objective function, controlled by the parameter *C*, which allows some data points to fall within the margin or to be misclassified.

(3) $$\begin{array}{c} \mathrm{Minimize}:\;\frac{1}{2}{∥w∥}^{2}+C\sum_{i=1}^{n}\varepsilon_{i} \end{array}$$

where ${∥w∥}^{2}$ represents the magnitude of the vector of weights, controlling the width of the margin, and $\varepsilon_{i}$ is the slack variable that allows some points to fall within the margin or to be misclassified.

Also, this SVM model employs kernel functions for non-linear problems. This approach facilitates classification by mapping the data to a higher dimensional space using kernel functions [25,26]. These kernels are defined by Equations (4)–(6).

(4) $$\begin{array}{cc} Polynomial\; & :K\left(X_{i},X_{j}\right)={\left(\gamma \left(X_{i},X_{j}\right)+r\right)}^{d} \end{array}$$

(5) $$\begin{array}{cc} Sigmoidal\; & :K\left(X_{i},X_{j}\right)=\tanh\left(\gamma \left(X_{i},X_{j}\right)+r\right) \end{array}$$

(6) $$\begin{array}{cc} Radial\;Basic\;Function\; & :K\left(X_{i},X_{j}\right)=exp(-\gamma ||X_{i}-X_{j}{\left|\right|}^{2}) \end{array}$$

where $K(X_{i},X_{j})$ is the kernel value between samples $X_{i}$ and $X_{j}$ and γ is the gamma hyperparameter, which is defined as the parameter controlling the influence of each training sample on the rest.

Finally, Equation (7) defines the decision function of the SVM model, which is based on the support vectors, which are the points that define the optimal margin. For a new sample *X*, the SVM classifies the sample according to the sign of the following function: if the value of the function is positive, the sample is classified as a class, and if it is negative, it is classified the other way around.

(7) $$\begin{array}{c} f\left(x\right)=sing(\sum_{i=1}^{n}\alpha_{i}y_{i}K\left(X_{i},X\right)+b) \end{array}$$

where $\alpha_{i}$ are the Lagrange coefficients associated with the support vectors [27], $y_{i}$ are the class labels (1 or 0), and $K(X_{i},X)$ is the Kernel value between the sample *X* and a support vector $X_{i}$.

#### 3.2.2. KNN Model

The fundamental principle of the KNN model [10] is that a sample is classified according to its nearest neighbors in the feature space, using Euclidean, Manhattan, or Minkowski metrics as described in Equations (8)–(10) in relation to [28]. The KNN model is initialized by its main parameters: “n_neighbors”, which represents the number k of nearest neighbor training data; “weights”, which defines the main strategies for prediction, either by uniform weighting or based on the distance to the neighbor; and “metric”, which determines the methodologies for calculating the separation between the test point and the training points.

(8) $$\begin{array}{cc} Euclidean\; & :d\left(X_{i},X_{j}\right)=\sqrt{\sum_{k=1}^{n}{\left(X_{ik}-X_{jk}\right)}^{2}} \end{array}$$

(9) $$\begin{array}{cc} Manhattan\; & :d\left(X_{i},X_{j}\right)=\sum_{k=1}^{n}\left|X_{ik}-X_{jK}\right| \end{array}$$

(10) $$\begin{array}{cc} Minkowski\; & :d\left(X_{i},X_{j}\right)=(\sum_{k=1}^{n}|X_{ik}-X_{jK}{{|}^{p})}^{1/p} \end{array}$$

Once the neighborhood for prediction has been determined [26], there are two main strategies. The new instance can be ranked according to the most frequent label in the neighborhood, where all neighbors vote with a weight of 1, a strategy known as uniform weights. However, in this case, the distance weights strategy is chosen to determine the final class, which is defined in Equation (11).

(11) $$\begin{array}{c} W_{i}=\frac{1}{d(X_{i},X)} \end{array}$$

where $W_{i}$ is the weight assigned to sample *i* and $d(X_{i},X)$ represents the Manhattan distance between sample *i* and the sample of interest *X*.

The decision function of the KNN model is defined in Equation (12). This Equation allows for the determination of which class this new sample belongs to, as the model queries its nearest neighbors and makes a decision based on a distance-weighted vote [28].

(12) $$\begin{array}{c} f\left(x\right)=arg\;max_{c}\;\sum_{i\in N_{k}}^{n}W_{i}\cdot 1\left(y_{i}=0\right) \end{array}$$

where $N_{k}$ is the set of *k* nearest neighbors, $y_{i}$ is the class of each neighbor, and 1 $\left(y_{i}=c\right)$ is the indicator function that takes the value one if the class $y_{i}$ belongs to class c and zero otherwise.

#### 3.2.3. RF Model

The RF model is based on the bagging technique (bootstrap aggregation) [24], where the parameters are initialized by variables such as “n_estimators”, which represents the number of trees the model builds; “max_depth”, which controls how many levels a decision tree can have before stopping the splitting process; “min_samples_split”, which controls the minimum number of samples a node must have before the tree splits it into smaller nodes; and “min_samples_leaf”, which represents the last node when a tree can no longer split. This tree is a structure that divides the data repository into subsets based on certain features using logical conditions at each node, generating rules such as if $X_{1}<v_{1}$ and $X_{2}<v_{2}$, then $\hat{y}=class$, where $X_{1}$ and $X_{2}$ are features of the dataset and $v_{1}$ and $v_{2}$ are threshold values. Spatial segmentation is performed using a criterion to determine whether a cut is appropriate. To do this, the notion of impurity is used to quantify how mixed the different classes are. There are two ways of doing this. The first is using the entropy defined in Equations (13) and (14), which is defined as [29]:

(13) $$\begin{array}{cc} Entropy: & \;\sum_{i=1}^{n}-P_{i}\cdot \log_{2}\left(P_{i}\right) \end{array}$$

(14) $$\begin{array}{cc} \; & P_{i}=\frac{\left|\{x\in c,\;clase(x)=1\}\right|}{\left|c\right|} \end{array}$$

For this assessment, the Gini impurity criterion defined in Equation (15) was chosen based on maximizing the impurity reduction.

(15) $$\begin{array}{c} Gini=1-\sum_{i=1}^{C}P_{i}^{2} \end{array}$$

where $P_{i}$ is the proportion of samples of class *i* at the current node and *C* is the total number of classes. The RF model has several parameters that are tuned to optimize its performance. The decision function for the RF model is defined as  

(16) $$\begin{array}{c} f\left(x\right)=mode\{T_{b}\left(x\right)\},\;b=1,2,3,...,B \end{array}$$

where $T_{b}\left(x\right)$ is the prediction of the *b*-th decision tree and n_estimators=b represents the number of decision trees the model builds and directly relates to the decision function. The final prediction of the model is determined by combining the predictions of multiple trees, where the class with the highest number of votes is chosen.

#### 3.2.4. GB Model

The principle is to build the GB model sequentially [30], fitting each new tree to the residual errors (the differences between the previous predictions and the true labels) [31]. The goal is to progressively correct the errors of the previous model using gradient descent techniques to optimize the loss function [4]. Then, the GB model calculates the final prediction by linearly combining the predictions of all the constructed trees, as shown in Equation (17).

(17) $$\begin{array}{c} F_{m}\left(x\right)=F_{m-1}\left(x\right)+\eta \cdot h_{m}\left(x\right) \end{array}$$

where $F_{m}\left(x\right)$ is the final prediction of the model after the *m*-th step, $F_{m-1}\left(x\right)$ is the cumulative prediction up to the previous step, η is the learning_rate hyperparameter that regulates the impact of each additional tree, and $h_{m}\left(x\right)$ represents the prediction function of the *m*-th tree. Then, to fit the model, the loss (error) function is minimized using the gradient descent technique. The model fits a tree at each iteration that predicts the correction direction needed to reduce the error [31] via the following equation.

(18) $$\begin{array}{c} h_{m}\left(x\right)=-\nabla L\left(y,F_{m-1}\left(x\right)\right) \end{array}$$

where $L(y,F_{m-1}\left(x\right))$ is the loss function to minimize, calculated based on the actual labels *y* and the cumulative predictions $F_{m-1}\left(x\right)$, and ∇ represents the loss gradient, which indicates how to adjust the model to improve accuracy. The overall model is the weighted sum of each decision tree [30], where the final prediction is determined by the linear combination of the outputs of each individual tree [31], adjusted by the learning rate.

#### 3.2.5. DT Model

The DT model is based on the separation of the dataset into more homogeneous subsets using decisions based on feature thresholds [24]. On the other hand, the DT model applies decision rules at each node, such as if $X_{1}<v_{1}$ and $X_{2}<v_{2}$, then $\hat{y}=class$, where $X_{1}$ and $X_{2}$ are features of the dataset and $v_{1}$ and $v_{2}$ are threshold values. DT has several hyperparameters that are used to adjust the performance and interpret the results, such as samples at Leaf Node (min_samples_leaf), Minimum Sample Split (min_samples_split), and Maximum Depth (max_depth); however, the Split Criterion is fundamental. For each node, the model evaluates the quality of the split through the criterion parameter, which can use either entropy or Gini impurity (13) and (15). The model builds a decision tree that minimizes the heterogeneity at each node, allowing accurate predictions on the test set [24].

The final prediction of the decision tree model is defined by the decision path in the tree, from the root to the leaf node, based on the input sample [29].

#### 3.2.6. NB Model

The NB model applies Bayes’ Theorem [32], assuming that all features are independent given the class outcome [4]. The naive independence assumption significantly simplifies the calculations. The equation for calculating the conditional probability that a sample *x* belongs to a class $C_{k}$ is expressed as   

(19) $$\begin{array}{c} P\left(C_{k}/X\right)=\frac{P\left(x/C_{k}\right)\cdot P\left(C_{k}\right)}{P(x)} \end{array}$$

where $P(C_{k}/X)$ is the posterior probability that *X* belongs to class $C_{k}$, $P(C_{k})$ is the a priori probability of class $C_{k}$, P(x) is the probability of the observed data *x*, and $P(x/C_{k})$ is the probability of observing data *X* given that it belongs to class $C_{k}$ (conditional probability).

The NB model assumes that all features $X=(x_{1},x_{2},\ldots ,x_{n})$ are independent, simplifying the equation as follows.

(20) $$\begin{array}{c} P\left(x/C_{k}\right)=\prod_{i=1}^{n}P\left(x_{i},C_{k}\right) \end{array}$$

In practice, P(X) is omitted because it is a constant for all classes and does not affect the final classification.

The conditional probability of a feature $x_{i}$, for which $x_{i}$ belongs to the class $C_{k}$ and in the case of Gaussian NB, is calculated by

(21) $$\begin{array}{c} P\left(x_{i}/C_{k}\right)=\frac{1}{\sqrt{2\pi \sigma_{K}^{2}}}exp\left(-\frac{\left(x_{i}-\mu_{k}^{2}\right)}{2\sigma_{K}^{2}}\right) \end{array}$$

where $\sigma_{K}^{2}$ is the hyperparameter var_smoothing, which is the variance of the feature in class $C_{k}$, and $\mu_{k}$ is the mean.

### 3.3. Proposed Rules-Based Diagnosis

The diagnosis stage is based on a rules-based approach [10] that relies on analyzing the behavioral patterns of the most significant variables of the system. Although the system monitors nine operational variables, the analysis of behavioral patterns reveals that some variables are critical for fault detection [14]. These variables are selected because they exhibit distinct patterns under different fault conditions, making them highly effective for accurate diagnosis.

The rules set specific thresholds and combinations of values that indicate the presence of a particular fault [14]. For example, if a variable exceeds or falls below a predefined threshold, it is classified as indicating a specific fault. Furthermore, the system evaluates the selected variables by comparing the measured values with the established rules and assigns a corresponding diagnosis. So, when a given observation meets the predefined criteria, the system classifies it as a particular type of failure. This structured methodology improves interpretability and ensures that the diagnosis is both accurate and directly linked to the physical behavior of the system.

## 4. Results

This section presents the main results obtained from the models described in the previous sections. It summarizes the operation of the cooling system, the feature selection process used for the classification methods, the model evaluation results, the fault detection in offline mode, and the rule-based diagnosis in online mode.

In addition, the hardware, software, and tools used to design and implement the proposed system are detailed based on [15]. The system uses a Raspberry Pi 4 Model B (Manufacturer: Raspberry Pi Foundation; Cambridge, UK), equipped with a Broadcom BCM2711 chipset and a quad-core ARM Cortex-A72 processor (Manufacturer: Broadcom Inc.; San Jose, CA, USA). Data acquisition is performed by an Arduino Nano (Manufacturer: Arduino AG; Boston, MA, USA), which features an ATmega328P microcontroller (Manufacturer: Microchip Technology Inc.; Chandler, AZ, USA), 2 KB of RAM, 32 KB of flash memory, and a 10-bit analog-to-digital converter (ADC). The instrumentation system consists mainly of five types of sensors selected for their accuracy, compatibility, and relevance to HVAC applications. The ACS712-30A Hall-effect sensor measures compressor current, offering electrical isolation and a range of ±30 A with analog output. The ZMPT101B module measures AC voltage by electromagnetic induction, providing a stable sinusoidal analog output and high accuracy in voltage detection. Two types of temperature sensors were implemented: the MLX90614, an infrared sensor capable of non-contact surface temperature measurement, and the DS18B20, a single-wire digital sensor for internal air temperature readings with a 0.5 °C resolution. The piezoresistive sensor SPKT0043P, capable of reading up to 500 psi with high stability, was implemented for pressure measurement. The system was programmed and executed with Python 3.11.9, Scikit-learn for ML implementations, and MySQL for structured data storage. Circuit simulation and PCB design were performed with EasyEDA, and signal analysis was performed with the support of Wolfram Mathematica 12.3.

The final prototype is shown in Section 4.6.

### 4.1. Dataset and Parameter

**Figure 7:**
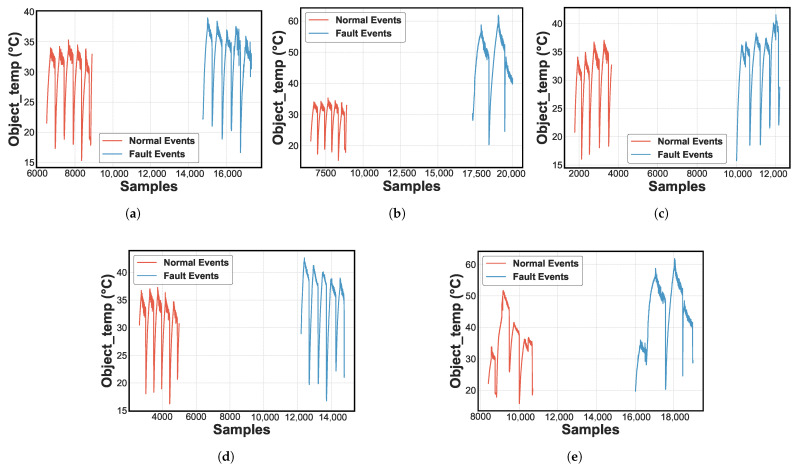
Compressor temperature evolution for (**a**) RO fault. (**b**) RU fault. (**c**) LR fault. (**d**) CA fault. (**e**) EA fault.

The analysis of the causes, characteristics, and consequences of each failure is performed to explain the dataset of the cooling system, i.e., the interaction of the parameters. Figure 7 shows the evolution of the characteristics of the five types of faults evaluated [3,25]. Specifically, Figure 7a shows an RO failure. Figure 7b shows an RU failure. Figure 7c shows a liquid LR failure. Figure 7d shows a CA failure. Figure 7e shows an EA failure [11]. The blue line represents normal operation, and the orange represents failure events. Specifically, the compressor temperature (°C) is selected as the main variable due to its ability to differentiate between normal and fault conditions. Also, a differentiated increase in the temperature level related to each failure pattern is observed.

**Figure 8:**
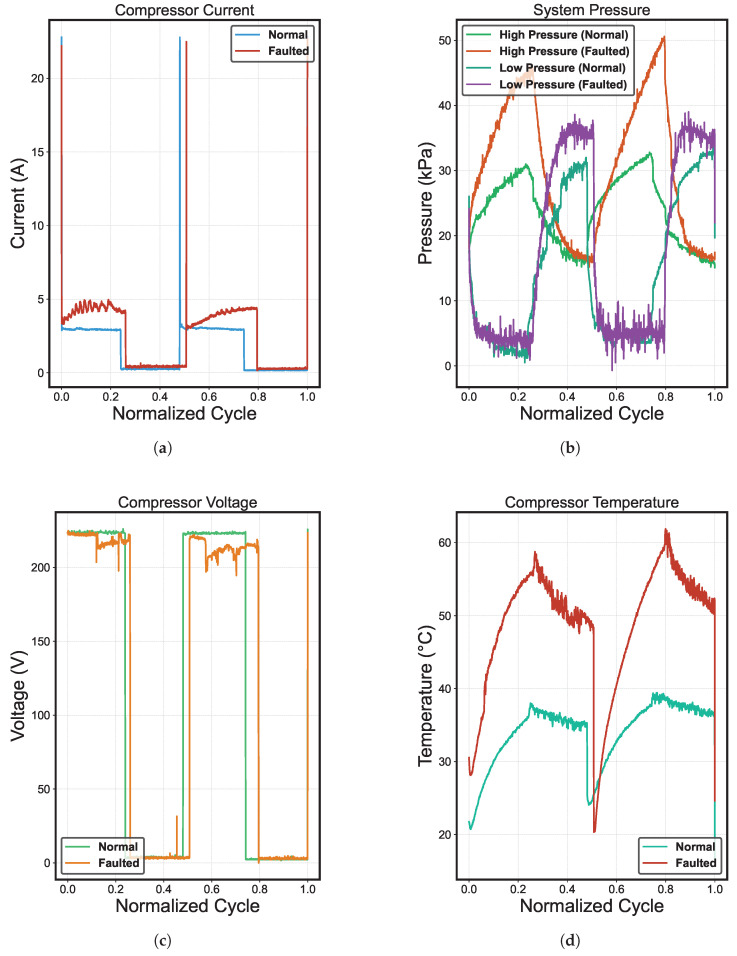
Comparison between normal and failed operation for the most important variables. (**a**) Compressor current. (**b**) System pressure. (**c**) Compressor voltage. (**d**) Compressor temperature.

Figure 8 shows the current, voltage, temperature, and pressure behavior as a function of the normalized cycles [25]. We can observe two complete cycles of operation of the compressor system in each result. The first was in normal conditions, and the other failed due to refrigerant undercharge. Also, the results show distinct patterns that are characteristic of critical failure. The refrigerant undercharge causes high voltage levels while the current remains unusually low, indicating that the compressor operates without adequate load. In addition, the temperature experiences a sustained rise, which is evidence of circuit overheating due to insufficient refrigerant to dissipate the generated heat. Although the pressure appears normal in terms of absolute values, its dynamic behavior deviates from the standard pattern.

### 4.2. Feature Selection

**Figure 9:**
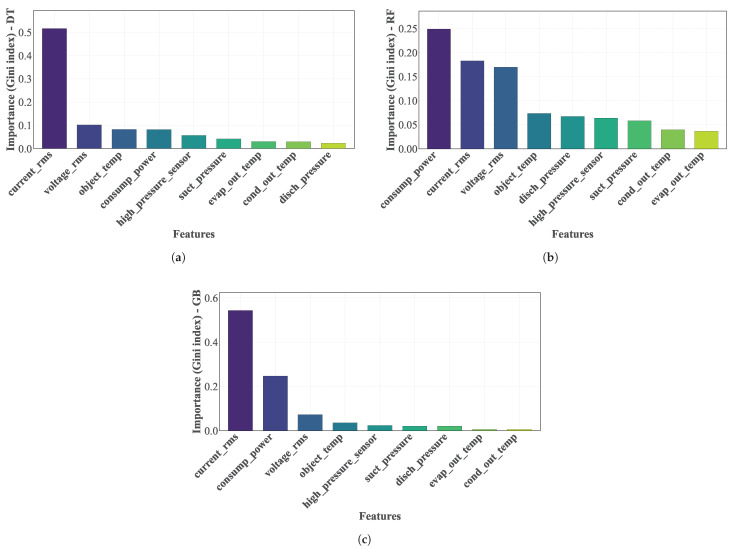
Representation of the importance of the variables for the DT, RF, and GB methods: (**a**) Importance of variables according to the Decision Tree. (**b**) Importance of variables according to the Random Forest. (**c**) Importance of Variables according to Gradient Boosting.

Figure 9 shows the importance of the variables in the classification task [11]. Figure 9a shows the importance of the variables according to the DT, Figure 9b shows the importance of variables according to the RF, and Figure 9c shows the importance of variables according to GB. The importance (reduction in the Gini index [23]) calculated from the split on a given predictor variable, averaged over all trees, shows that a high value in the index indicates an important predictor variable. So, for the classification methods DT, RF, and GB [10], the variables Tcomp, Icomp, Vcomp, and Wcomp [11,14] are the most important predictors in the FDD process. However, there is no clear decrease in the importance of differentiating essential from non-essential predictors.

### 4.3. Evaluation of Models

The models were evaluated through a dataset that was systematically divided into three subsets [25]—training (50%), validation (44%), and testing (6%)—ensuring a robust evaluation of the performance of each model. In addition, calculation of the accuracy (Equation (22)), precision (Equation (23)), sensitivity (Equation (24)), and specificity (Equation (25)) of the model results based on [33] was necessary to complete the analysis of these models.

(22) $$\begin{array}{cc} Accuracy & =\frac{TP+TN}{TP+TN+FP+FN} \end{array}$$

(23) $$\begin{array}{cc} Precision & =\frac{TP}{TP+FP} \end{array}$$

(24) $$\begin{array}{cc} Sensitivity & =\frac{TP}{TP+FN} \end{array}$$

(25) $$\begin{array}{cc} Specificity & =\frac{TN}{TN+FP} \end{array}$$

**Figure 10:**
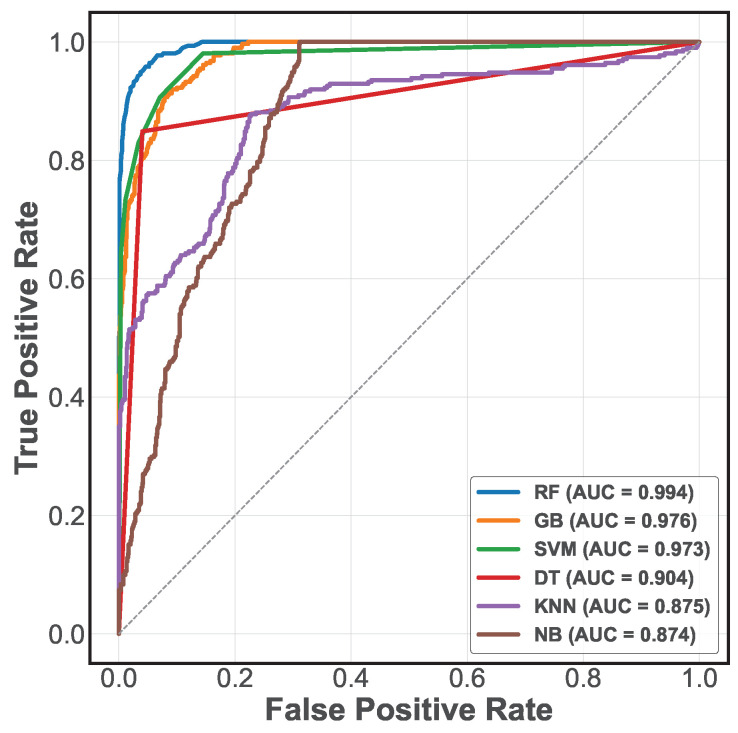
ROC curve for different models.

Equations (22)–(25) provide the comprehensive evaluation of the performance of the classification models [11], where TP represents true positives, TN true negatives, FP false positives, and FN false negatives. In addition, the construction of Receiver Operating Characteristic (ROC) curves [4] is performed to better understand the classification models’ performance. Figure 10 presents the ROC curves of the six models evaluated, showing their discriminative ability at different decision thresholds. These curves represent the trade-off between sensitivity (true positive rate) and specificity (false positive rate), giving a detailed insight into the behavior of each model under different classification conditions. In addition, the area under the ROC curve (AUC-ROC) quantifies the overall discriminative ability of the models, where a value closer to 1 indicates superior classification performance.

**Figure 11:**
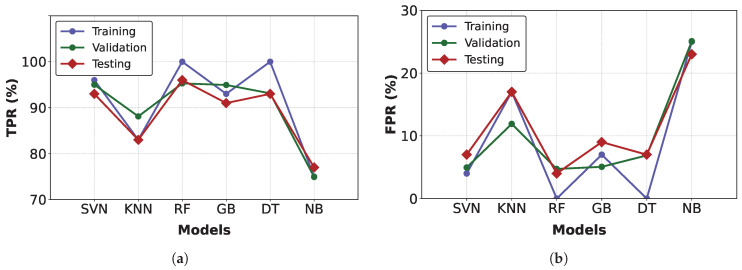
TPR and FPR values for each classification method. (**a**) TPR (%). (**b**) FPR (%).

On the other hand, Figure 11 presents the TPR and FPR values [4] at different threshold points for the three subsets evaluated. These results demonstrate robust and reliable fault detection in PAC systems.

### 4.4. Fault Detection Result in Offline Mode

To validate the importance of preprocessing, the performance of the models evaluated with and without the application of the RUS module was compared. Table 6 shows the results. An improvement in accuracy and sensitivity is observed when balancing is applied, confirming the positive impact of preprocessing.

**Figure 12:**
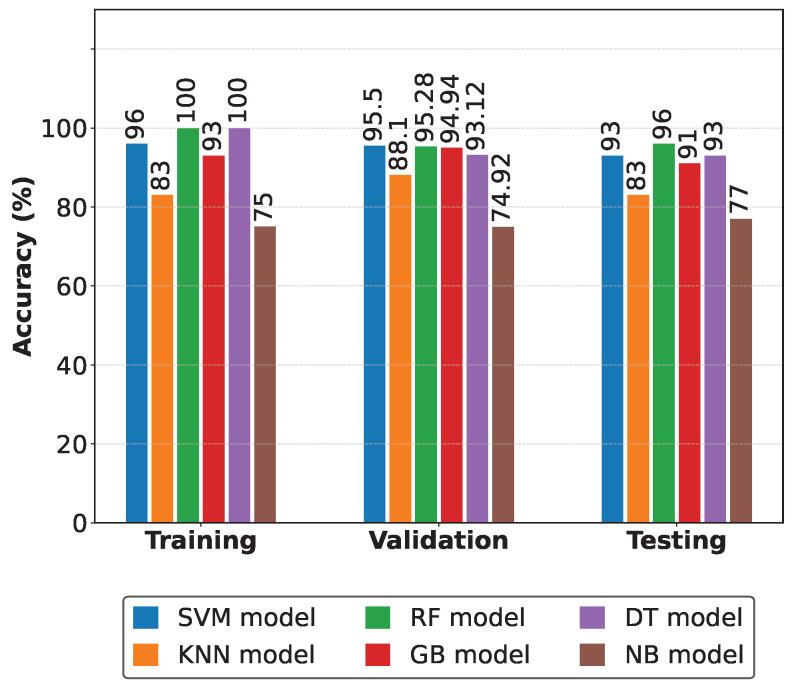
Comparison of the detection models’ evaluation metrics (accuracy).

Figure 12 compares the evaluation metrics of the detection models [33]. In this comparison, the consistent superiority of the Random Forest classifier in all evaluation phases is highlighted. Furthermore, this comparative analysis evaluates the effectiveness of the proposed FDD system. The model selection with the best conditions (metrics) for fault detection is based on tables and graphs [3]. So, to obtain our results, we applied each classification method to our generated database. This dataset was divided into three subsets—training, validation, and testing—which allowed a thorough evaluation of the performance of each model at different stages. The main metric used for comparison was accuracy, as this directly measures the model’s ability to correctly classify both normal and faulty conditions. Furthermore, in previous studies on fault detection in cooling units, researchers have used accuracy as a key indicator due to its interpretability and relevance in classification tasks. For another data configuration (preprocessed), other levels of accuracy were observed. When the amount of data increases, the accuracy levels vary, but in the order of 0.125 or a maximum of 2 to 3%.

Table 7 summarizes the hyperparameter optimization process [14] for each model. The optimal values of these parameters were determined using cross-validation (CV) techniques [4]. The selected hyperparameters for each classification method were tuned to maximize model performance. This systematic optimization process was implemented using Scikit-learn’s GridSearchCV functionality, which improved the evaluation metrics, namely the accuracy during the offline validation phase. The most suitable configurations for each model were determined through iterative training and validation of the models on different subsets of the dataset. This approach not only improved the accuracy levels of the models but also prepared them for reliable implementation in the real-time fault detection system.

The variables shown in the hyperparameter column in Table 7 are described in Section 3.2. Specifically, Equations (1)–(21) describe how these variables interact.

**Figure 13:**
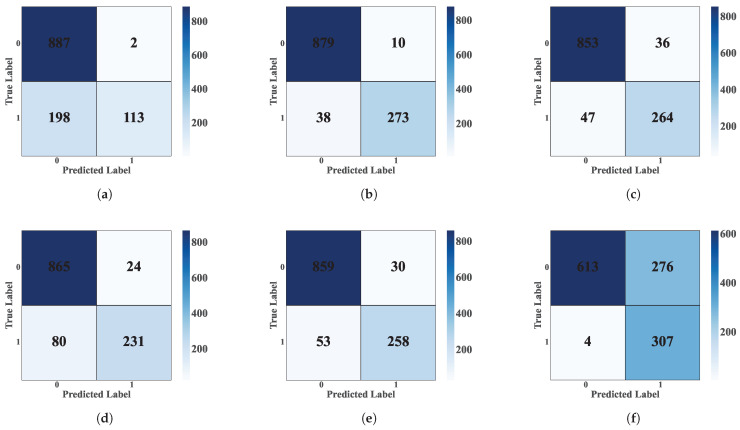
Confusion matrices for the models. (**a**) Confusion Matrix—SVM. (**b**) Confusion Matrix—RF. (**c**) Confusion Matrix—DT. (**d**) Confusion Matrix—GB. (**e**) Confusion Matrix—KNN. (**f**) Confusion Matrix—NB.

Figure 13 shows the confusion matrices for each classification model [19]. These matrices, generated from the database’s test subset, allow the models’ performance in fault detection [23] to be evaluated in offline mode [10]. Each matrix cell represents the number of predictions for a given class, where the main diagonal indicates correct classifications and off-diagonal cells represent incorrect classifications [19].

Table 8 summarizes the comparative analysis of the evaluation metrics [33]. This analysis allowed the selection of the ML model with the best performance in fault event detection [4]. The models were evaluated against key metrics such as accuracy, precision, specificity, false positive rate (FPR), and true positive rate (TPR). The RF model demonstrated the best performance, achieving an accuracy of 95.75%, the highest among all classifiers evaluated. In addition, this model exhibited an excellent balance between accuracy (95.74%) and sensitivity (98.65%), with a relatively low FPR of 12.54%. On the other hand, the SVM model performed with an accuracy of 93.08%, closely followed by the DT model with 93.83%. Although the GB model achieved an accuracy of 91.33%, its performance was lower than that of the other models. On the other hand, the KNN and NB algorithms presented limitations, with accuracies of 83.33% and 76.67%, respectively. We can note that the NB model showed the highest overall classification accuracy (99.35%) but the lowest sensitivity (68.96%), indicating its tendency to minimize false positives at the cost of omitting real detections. This comprehensive evaluation confirms that the RF model provides the best balance between the various performance metrics for fault detection in PAC systems.

### 4.5. FDD Result Online Mode

In Figure 7, unique patterns of signal variation were observed for each type of fault [3]. High voltage levels, low current, normal pressure, and elevated temperature are observed for refrigerant undercharge, indicating poor compression and circuit overheating. In RO faults, voltage levels are normal. However, current, pressure, and temperature are high and there is a risk of freezing and prolonged cycling. So, in liquid line restrictions, the variables are normal except for high temperature, which shows similar behavior. On the other hand, in failures due to reduced airflow in the condenser or evaporator, combinations of high or low voltage with irregular current and high temperatures were detected, showing overheating and altered cooling times.

Table 9 shows the classification rules derived from the analysis based on what was described in Section 4.2. We can observe the identification of the four key variables most relevant in the FDD process: Tcomp, Icomp, Vcomp, and Wcomp [14,34]. The rules indicate that Tcomp plays a crucial role in differentiating between the different failure modes, with further refinement provided by Icomp, Vcomp, and Wcomp. Furthermore, the most influential variable does not always correspond to the first division in decision making, as the latter is determined by information gain rather than absolute importance. Therefore, this classification framework ensures reliable real-time fault detection based on sensor data.

The results obtained from tests in both delayed (database) and real-time conditions validated the effectiveness of early fault detection. The RF model stood out for its superior performance, achieving 96% accuracy in the delayed condition and 95.28% in the real-time test. The accuracy of 95.31% indicates that, when predicting a positive class, the model has a high probability of being correct, thus reducing false positives. Sensitivity, with a value of 81.87%, quantifies the model’s ability to detect real failures, minimizing false negatives. Specificity, which reached 98.9%, indicates correctly identifying fault-free instances and avoiding false positives, as shown in Table 10. Finally, the analysis is observed in the real-time validation test (P-V), simulating the system’s real behavior together with a constant heat injector element (heater).

### 4.6. Final Prototype

**Figure 14:**
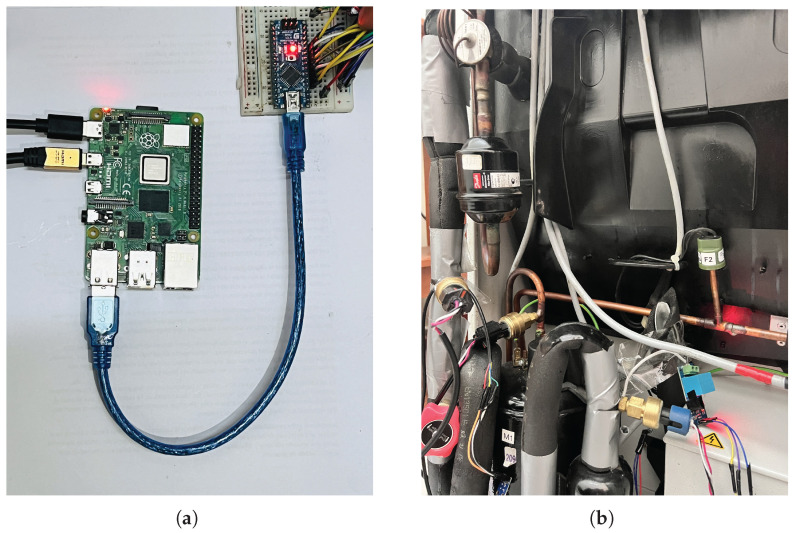
The final prototype integrates (**a**) a microprocessor (Raspberry Pi) connected via USB to a microcontroller (Arduino Nano). (**b**) Installation and location of the sensors.

The final prototype developed and implemented for the study is shown in Figure 14.

## 5. Discussion

The results obtained align with previous research on data-driven FDD methodologies for cooling systems. Ebrahimifakhar et al. [10] demonstrated the effectiveness of ML classification models for FDD in rooftop units by evaluating multiple algorithms from SVM, RF, and GB models to diagnose faults such as compressor valve leakage (VL), RU, RO, RL, CA, EA, and the presence of noncondensable (NC) gases. Their study highlighted the high classification accuracy of SVM (96.2%). In comparison, models such as Linear Discriminant Analysis performed worse (76.2%), indicating that the choice of ML model significantly influences the reliability of fault detection. Our research similarly implemented a multi-class classification approach, training six ML models, SVM, RF, GB, GB, DT, KNN, and NB, using key operational variables (pressure, temperature, current, and voltage). Consistent with previous work, the RF model outperformed the other classifier models, achieving 96% accuracy, while the NB model exhibited the worst performance (77%). These results reinforce the effectiveness of ensemble-based methods for handling non-linear relationships and complex failure patterns in refrigeration units. In addition, data preprocessing was key in improving model performance. On the other hand, in [3], the authors have emphasized that outlier removal and transient behavior analysis are essential to improve accuracy in FDD, an approach we incorporated using RUS to balance the dataset.

Our results agree with the results obtained in [5], which explored non-intrusive load monitoring (NILM) for fault detection in HVAC systems using electrical measurements (voltage and current). While NILM provides a complementary approach to sensor-based FDD, our results suggest that direct sensor measurements allow for higher classification accuracy due to their ability to capture real-time fluctuations in system behavior. The integration of real-time data processing and ML models offers a robust and scalable solution for fault diagnosis in PAC systems, addressing a key gap in the existing methodologies. Also, Table 11 shows a concrete comparison between the previously mentioned research results and our results. This comparison is based on five aspects: the faults evaluated, the methods used, the variables considered in the research, the data source, and the experimental validation. We can observe that for the experimental validation in [10], the results do not have real tests, while in [2] the authors do not have results in a real system. Likewise, in [5], the experimental validation of the results was via analysis methods without HVAC intervention, and in [3], the results do not have controlled experimental validation. In comparison, our results had full experimental validation, i.e., in a real system and full experimental validation.

Finally, the superior performance of the RF model can be attributed to its ability to handle non-linear relationships, its tolerance to overfitting, and its robustness to noise in the data. Its assembly mechanism, which is achieved through bagging and random feature selection, allows it to capture complex patterns. In contrast, models such as KNN or NB are sensitive to scaling and variable redundancy, negatively affecting their performance. In environments with high-class separation, models such as SVM could provide similar or even better results. However, RF demonstrated better stability and accuracy in real-world conditions with non-linear and noisy data. Therefore, the performance of models is a function of the type and amount of data. We also sought to improve the performance through a preprocessing step.

## 6. Conclusions

This study developed an automatic real-time FDD system that classifies events as faulty or normal by analyzing status signals. The analyzed models are based on data and ML, where a specific strategy is proposed for five types of system faults. Firstly, a comprehensive database was developed to train FDD models under specific fault conditions, allowing the analysis of critical status signals such as pressure, temperature, current, and voltage. Secondly, a real-time FDD system that leverages ML algorithms to detect and diagnose faults with high accuracy and reliability was implemented. Through a comparative evaluation of six ML classification models (SVM, KNN, DT, GB, NB, and RF), the RF model was found to be the most effective, achieving 96% accuracy in offline evaluation. This model demonstrated superior performance in handling non-linear relationships and complex fault patterns. In addition, real-time validation of the RF model on the SK3328.500 system achieved an accuracy of 95.28%. An additional validation test under real-world conditions for the PAC system confirmed an accuracy of 93.49%, with the optimal hyperparameters set to max_depth = 25 and n_estimators = 150. We conclude that the most influential predictor variables in fault classification were Tcomp, Icomp, Vcomp, and Wcomp, with decision rules that allowed the accurate differentiation of fault types. Despite some false positives and negatives, the system effectively detected and classified key fault scenarios, including RU, RO, RL, CA, and EA.

Finally, the use of paired t-tests and Wilcoxon tests confirmed that the Random Forest model consistently outperformed all other classifiers, with p-values less than 0.05. This constitutes statistical evidence of the robustness and accuracy of the proposed failure detection system.

## 7. Future Work

This research opens several avenues for future investigation. First, the current FDD framework focuses on five predefined fault types. Therefore, future work could evaluate additional classifications when incorporating new types of faults, sensors, or different operating conditions and include gradual degradation patterns. These evaluations would be performed to improve the adaptability and generalizability of the system in the different HVAC configurations of industrial environments without the need to redesign the base model completely. Furthermore, the integration of new sensing modalities, such as frequency signals, vibration, or infrared thermal imaging. It could provide complementary information that improves diagnostic accuracy. Further improvements could involve exploring advanced signal preprocessing techniques and hybrid learning models that combine classical ML with deep learning methods to capture more complex fault behaviors. Including additional operational variables may also improve feature richness and model robustness. As embedded hardware continues to evolve, implementing this FDD framework on next-generation edge devices with greater computational power and energy efficiency, such as NVIDIA Jetson, Coral TPU, or newer versions like ORANGE PI, will enable more agile and scalable real-time fault detection solutions in industrial environments. Finally, future work should develop an analysis of the results to correlate these results with physical implications on the system under evaluation.

## Figures and Tables

**Table 1:** Specifications of the PAC system.

System Type	Nominal Capacity (kW)	Refrigerant	Expansion Device	Compressor Type	Operating Mode
PAC	2	R134	Fixed orifice	Scroll	Cooling

**Table 2:** Five faults and fault descriptions.

Fault Type	Abbreviation	Description
Refrigerant undercharge	RU	The refrigerant charge level is lower than recommended (65%—618 g)
Refrigerant overcharge	RO	The refrigerant charge level is higher than recommended (130%—1235 g)
Line restriction	RL	Implemented by a regulating valve located near the compressor panel to reduce pressure loss
Condenser airflow reduction	CA	Implemented by blocking a portion of the surface (except where the surface is already partially blocked)
Evaporator airflow reduction	EA	Implemented by blocking the evaporator outlet filter with a mesh to reduce air flow

**Table 3:** The statistics of the input variables of the total dataset.

Input Variable	Unit	Mean	Minimum	Maximum
Tcomp (T)	°C	35	15	60
Icomp (A)	A	3	2.5	23
Vcomp (V)	V	222	215	229
Phightpres (Ph)	kPa	100	65	190
Psuc (Ps)	kPa	40	1	65
Pdtsc (Pd)	kPa	100	65	190
Jevap (Te)	°C	25	−3	19
Tcond (Tc)	°C	25	18	35
Wcomp (W)	W	680	600	1250

**Table 4:** Description of the confusion matrix for the detection result.

		Predicted Class	
		**Fault**	**Normal**
**True Class**	Fault	TP	FN
	Normal	FP	TN

**Table 5:** A full list of SKLearn packages and functions used.

Classification Method	Function	Computational Complexity	Parallelization	Handling Missing Values	Sensitivity to Feature Scaling
SVM	SVC()	O(n²) − O(n³)	Limited	Requires preprocessing	High (requires normalization)
KNN	KNeighborsClassifier()	O(n·d) for prediction	Good for prediction	Sensitive (requires imputation)	High (requires normalization)
RF	RandomForestClassifier()	O(n·log(n)·m)	Excellent	Robust	Low (invariant to scaling)
DT	DecisionTreeClassifier()	O(n·log(n))	Limited	Robust	Low (invariant to scaling)
GB	GradientBoostingClassifier()	O(n·log(n)·m)	Limited (sequential)	Moderately robust	Low (invariant to scaling)
NB	GaussianNB()	O(n·d)	Good	Requires preprocessing	Low (but assumes normal distribution)

**Table 6:** Performance of the models evaluated with and without the application of the RUS module.

	With RUS		Without Preprocessing	
	**Accuracy (%)**	**Sensitivity (%)**	**Accuracy (%)**	**Sensitivity (%)**
Modelo SVM	96	98	87	90
Modelo RF	93	96	85	88
Modelo DT	93	96	86	88
Modelo GB	91	97	82	88
Modelo KNN	83	99	69	80
Modelo NB	77	68	60	68

**Table 7:** The list of tuning parameters for each classification method.

Models	Hyperparameters	Range of Values	Optimal Values
SVM	C,	[400, 450, 500, 550, 600, 700, 800, 850, 900, 950, 1000, 1150]	900
	gamma	[0.1, 0.2, 0.3, 0.9, 0.01, 0.02, 0.03, 0.09, 0.001, 0.002, 0.009]	0.01
RF	Min_samples_split,	[0.5, 1, 2, 3, 4, 5]	1
	min_samples_leaf,	[0.5, 1, 2, 3, 4, 5]	2
	max_depth,	[None, 10, 15, 20, 25, 30, 35, 40, 45, 50]	25
	n_estimator	[100, 150, 200, 250, 300, 350, 400, 450, 500]	150
KNN	n_neighbors,	[1, 3, 5, 7, 9, 25, 35, 45, 55, 65, 75, 85, 95]	3
	metric,	[“euclidean”, “manhattan”, “minkowski”]	manhattan
	weights	[“uniform”, ”distance”]	distance
DT	Criterion,	[“gini”, “entropy”]	Gini
	max_depth,	[None, 10, 20, 30]	None
	min_samples_split,	[2, 5, 10]	1
	min_samples_leaf	[1, 2, 4]	2
NB	var_smoothing	[$1\times 10^{-9}$, $1\times 10^{-8}$, $1\times 10^{-7}$, $1\times 10^{-6}$, $1\times 10^{-5}$, $1\times 10^{-4}$, $1\times 10^{-3}$, $1\times 10^{-2}$, $1\times 10^{-1}$]	0.1
GB	Learning_rate,	[0.01, 0.05, 0.1, 0.2, 0.3]	0.3
	n_estimators,	[50, 100, 150, 200]	150
	max_depth,	[3, 4, 5, 6, 10]	6
	min_samples_split,	[2, 5, 10]	5
	min_samples_leaf	[1, 2, 4]	2

**Table 8:** Metrics for evaluating fault detection results in offline mode.

	Confusion Matrix				Accuracy	Precision	Sensitivity (TPR)	Specificity	FPR	Total Events
	**FP**	**FN**	**TP**	**TN**						
Modelo SVM	53	30	859	258	93.08	94.18	96.62	82.96	17.04	1200
Modelo RF	39	12	877	272	95.75	95.74	98.65	87.46	12.54	1200
Modelo DT	40	34	855	271	93.83	95.53	96.18	87.14	12.86	1200
Modelo GB	80	24	865	231	91.33	91.53	97.30	74.27	25.73	1200
Modelo KNN	198	2	887	113	83.33	81.75	99.78	36.35	63.65	1200
Modelo NB	4	276	613	307	76.67	99.35	68.96	98.71	1.29	1200

**Table 9:** Rules for fault classification.

Rule	Condicions	Label
1	If 20 °C≤Tcomp≤60 °C, 3.3A≤Icomp≤4.2A, 220V≤Vcomp≤222V, 10psi≤Wcomp≤50psi, and 5psi≤low_pressure_sensor≤40psi	“Falla OC”
2	If 20 °C≤Tcomp≤38 °C, 2.8A≤Icomp≤3.1A, 220V≤Vcomp≤222V, 10psi≤Wcomp≤28psi, and 2psi≤low_pressure_sensor≤27psi	“Falla UC”
3	If 20 °C≤Tcomp≤80 °C, 3A≤Icomp≤3.4A, and 220V≤Vcomp≤224V	“Falla RL”
4	If 20 °C≤Tcomp≤40 °C, 3.1A≤Icomp≤3.4A, and 221V≤Vcomp≤224V	“Falla CA”
5	If 25 °C≤Tcomp≤48 °C, 3A≤Icomp≤4.8A, 214V≤Vcomp≤219V, and 10psi≤Wcomp≤40psi	“Falla EA”

**Table 10:** Metrics for evaluating fault detection results in online mode.

	Confusion Matrix				Accuracy	Precision	Sensitivity	Specificity	FPR	Total Events
	**FP**	**FN**	**TP**	**TN**						
Modelo RF	12	54	244	1090	0.9528	0.9531	0.8187	0.9891	0.0108	1400
Modelo P-V	40	71	1061	544	0.9349	0.9633	0.9367	0.9315	0.068	1706

**Table 11:** Comparison of research on methods used.

Reference	Faults Evaluated	Methods Used	Variables Considered	Source of Data	Experimental Validation
Ebrahimifakhar et al. [10]	7 faults on RTU	Bagging, XGBoost, LDA, QDA, KNN, RF, AdaBoost, SVM, LR	15 variables (some artificial)	Simulated base + SMOTE	Without real tests
Zhu et al. [2]	2 faults on refrigerant	GBDT + semi-empirical model	10 variables (energy consumption)	Experimental data	Without real system tests
Laughman et al. [5]	6 faults on HVAC systems	NILM (Electrical Signal Analysis)	Voltage, current, electrical transients	Electrical signals	Analysis methods without HVAC intervention
Our research	5 faults on precision systems	SVM, KNN, DT, GB, RF, NB	10 variables (temperature, voltage, current, pressure)	Own base with real tests	**Full experimental validation**
Wang et al. [3]	9 faults on VRF	K-Means, DBSCAN, Bayesian networks	12 variables (compressor efficiency, refrigerant flow)	Real VRF data	Without controlled experimental validation

## Data Availability

Data is contained within the article.

## Abbreviations

The following abbreviations are used in this manuscript:

HVAC	Heat, ventilation, and air conditioning
PAC	Precision air conditioning
FDD	Fault detection and diagnosis
ML	Machine learning
SVM	Support Vector Machine
KNN	K-Nearest Neighbors
RF	Random Forest
GB	Gradient Boosting
DT	Decision Tree
NB	Naïve Bayes
CSV	Comma-separated values
RO	Refrigerant overcharge
RU	Refrigerant undercharge
RL	Line restriction
CA	Condenser airflow reduction
EA	Evaporator airflow reduction

## References

[1] Chen J., Zhang L., Li Y., Shi Y., Gao X., Hu Y. (2022). A review of computing-based automated fault detection and diagnosis of heating, ventilation and air conditioning systems. Renew. Sustain. Energy Rev..

[2] Zhu X., Du Z., Chen Z., Jin X., Huang X. (2019). Hybrid model based refrigerant charge fault estimation for the data centre air conditioning system. Int. J. Refrig..

[3] Wang Y., Li Z., Chen H., Zhang J., Liu Q., Wu J., Shen L. (2021). Research on diagnostic strategy for faults in VRF air conditioning system using hybrid data mining methods. Energy Build..

[4] Kim I., Kim W. (2021). Development and validation of a data-driven fault detection and diagnosis system for chillers using machine learning algorithms. Energies.

[5] Armstrong P.R., Laughman C.R., Leeb S.B., Norford L.K. (2006). Detection of rooftop cooling unit faults based on electrical measurements. HVAC&R Res..

[6] Gao L., Li D., Li D., Yao L., Liang L., Gao Y. (2019). A novel chiller sensors fault diagnosis method based on virtual sensors. Sensors.

[7] Garreta R., Moncecchi G. (2013). Learning Scikit-Learn: Machine Learning in Python.

[8] Breiman L. (2001). Random forests. Mach. Learn..

[9] Xu S., Zhao Q., Yin K., Zhang F., Liu D., Yang G. (2019). Combining random forest and support vector machines for object-based rural-land-cover classification using high spatial resolution imagery. J. Appl. Remote Sens..

[10] Ebrahimifakhar A., Kabirikopaei A., Yuill D. (2020). Data-driven fault detection and diagnosis for packaged rooftop units using statistical machine learning classification methods. Energy Build..

[11] Tun W., Wong J.K.W., Ling S.H. (2021). Hybrid random forest and support vector machine modeling for HVAC fault detection and diagnosis. Sensors.

[12] Wen J., Li S. (2011). Tools for Evaluating Fault Detection and Diagnostic Methods for Air-Handling Units.

[13] Liao H., Cai W., Cheng F., Dubey S., Rajesh P.B. (2021). An online data-driven fault diagnosis method for air handling units by rule and convolutional neural networks. Sensors.

[14] Wang J., Li G., Chen H., Liu J., Guo Y., Hu Y., Li J. (2017). Liquid floodback detection for scroll compressor in a VRF system under heating mode. Appl. Therm. Eng..

[15] Zhang M., Xing X., Wang W. (2023). Smart Sensor-Based Monitoring Technology for Machinery Fault Detection. Sensors.

[16] Chiang L.H., Russell E.L., Braatz R.D. (2000). Fault Detection and Diagnosis in Industrial Systems.

[17] Fernández-Delgado M., Cernadas E., Barro S., Amorim D. (2014). Do we need hundreds of classifiers to solve real world classification problems?. J. Mach. Learn. Res..

[18] Bishop C.M., Nasrabadi N.M. (2006). Pattern Recognition and Machine Learning.

[19] Meas M., Machlev R., Kose A., Tepljakov A., Loo L., Levron Y., Petlenkov E., Belikov J. (2023). Explainability and Transparency of Classifiers for Air-Handling Unit Faults Using Explainable Artificial Intelligence (XAI). Sensors.

[20] Demidova L., Klyueva I., Pylkin A. (2019). Hybrid approach to improving the results of the SVM classification using the random forest algorithm. Procedia Comput. Sci..

[21] Li J., Guo Y., Wall J., West S. (2019). Support vector machine based fault detection and diagnosis for HVAC systems. Int. J. Intell. Syst. Technol. Appl..

[22] Yan R., Ma Z., Zhao Y., Kokogiannakis G. (2016). A decision tree based data-driven diagnostic strategy for air handling units. Energy Build..

[23] Zhou Z., Li G., Wang J., Chen H., Zhong H., Cao Z. (2023). A comparison study of basic data-driven fault diagnosis methods for variable refrigerant flow system. Energy Build..

[24] Maroco J., Silva D., Rodrigues A., Guerreiro M., Santana I., de Mendonça A. (2011). Data mining methods in the prediction of Dementia: A real-data comparison of the accuracy, sensitivity and specificity of linear discriminant analysis, logistic regression, neural networks, support vector machines, classification trees and random forests. BMC Res. Notes.

[25] Mattera C.G., Quevedo J., Escobet T., Shaker H.R., Jradi M. (2021). A Method for Fault Detection and Diagnostics in Ventilation Units Using Virtual Sensors. Sensors.

[26] Beghi A., Brignoli R., Cecchinato L., Menegazzo G., Rampazzo M. (2015). A data-driven approach for fault diagnosis in HVAC chiller systems. Proceedings of the 2015 IEEE Conference on Control Applications (CCA).

[27] Awad M., Khanna R., Awad M., Khanna R. (2015). Support vector regression. Efficient Learning Machines: Theories, Concepts, and Applications for Engineers and System Designers.

[28] Vapnik V. (1998). Statistical Learning Theory.

[29] Biau G., Scornet E. (2016). A random forest guided tour. Test.

[30] Chen T., Guestrin C. Xgboost: A scalable tree boosting system. Proceedings of the 22nd ACM Sigkdd International Conference on Knowledge Discovery and Data Mining.

[31] Chakraborty D., Elzarka H. (2019). Early detection of faults in HVAC systems using an XGBoost model with a dynamic threshold. Energy Build..

[32] Hartigan J.A. (2012). Bayes Theory.

[33] Nie L., Wu R., Ren Y., Tan M. (2023). Research on fault diagnosis of HVAC systems based on the ReliefF-RFECV-SVM combined model. Actuators.

[34] Li D., Hu G., Spanos C.J. (2016). A data-driven strategy for detection and diagnosis of building chiller faults using linear discriminant analysis. Energy Build..

